# Telomere length predicts timing and intensity of migratory behaviour in a nomadic songbird

**DOI:** 10.1098/rsbl.2022.0176

**Published:** 2022-08-03

**Authors:** Ben J. Vernasco, Heather E. Watts

**Affiliations:** ^1^ School of Biological Sciences, Washington State University, Pullman, WA, USA; ^2^ Center for Reproductive Biology, Washington State University, Pullman, WA, USA

**Keywords:** telomeres, facultative migration, Zugunruhe, nomad, ageing, state-dependent behavior

## Abstract

Our understanding of state-dependent behaviour is reliant on identifying physiological indicators of condition. Telomeres are of growing interest for understanding behaviour as they capture differences in biological state and residual lifespan. To understand the significance of variable telomere lengths for behaviour and test two hypotheses describing the relationship between telomeres and behaviour (i.e. the causation and the selective adoption hypotheses), we assessed if telomere lengths are longitudinally repeatable traits related to spring migratory behaviour in captive pine siskins (*Spinus pinus*). Pine siskins are nomadic songbirds that exhibit highly flexible, facultative migrations, including a period of spring nomadism. Captive individuals exhibit extensive variation in spring migratory restlessness and are an excellent system for mechanistic studies of migratory behaviour. Telomere lengths were found to be significantly repeatable (*R* = 0.51) over four months, and shorter pre-migratory telomeres were associated with earlier and more intense expression of spring nocturnal migratory restlessness. Telomere dynamics did not vary with migratory behaviour. Our results describe the relationship between telomere length and migratory behaviour and provide support for the selective adoption hypothesis. More broadly, we provide a novel perspective on the significance of variable telomere lengths for animal behaviour and the timing of annual cycle events.

## 1. Introduction

Animal behaviour can depend upon an individual's environment and physiology [1–3]. Such state-dependent expression of behaviour can maximize an individual's residual reproductive value [1,4]. Identifying state variables indicative of an organism's physiological condition is essential to understanding state-dependent animal behaviour [2,5]. Telomeres are one state variable of growing interest for understanding behaviour [6,7].

Telomeres are repetitive, non-coding segments of DNA found on the end of eukaryotic chromosomes [8]. Telomeres maintain chromosomal integrity and mediate cellular signalling processes [8,9]. Changes in telomere lengths (i.e. telomere dynamics) depend upon intrinsic and extrinsic processes [10–12] and are associated with health, individual quality and residual lifespan [13–19]. Telomeres are therefore considered a biomarker of biological state [8,17,20]. A number of studies also report correlations between telomere lengths and behaviour [6,20–23]. These relationships could arise due to specific behaviour causing changes in telomere lengths (i.e. causation hypothesis, [6]). Alternatively, individuals with certain telomere lengths may be more likely to express particular behaviour (i.e. selective adoption hypothesis, [6]) via direct effects (mediated by effects of telomeres on gene expression, [24]) or indirectly due to a correlation between telomeres, behaviour, and a third factor. The causation hypothesis predicts individuals should exhibit similar telomere lengths prior to expressing a specific behaviour whereas the selective adoption hypothesis predicts differences in telomere length before behaviour is adopted. Additionally, the causation hypothesis predicts differences in telomere dynamics between individuals adopting different behaviour, whereas selective adoption does not predict telomere dynamics to be associated with individual differences in behaviour [6]. Outside of humans, however, relatively few studies have assessed these non-mutually exclusive hypotheses using this framework (but see [19]).

Variation in migratory behaviour, including the timing of migration, is important as it can impact survival and reproductive success [25–29]. Studies have previously measured telomere dynamics to understand the consequences of variable migratory behaviour [18,30,31], but the extent to which telomere lengths predict subsequent migratory behaviour, including its timing, is not well understood. Studying variation in migratory behaviour, particularly in systems where migratory behaviour is highly flexible and under less rigid genetic control (e.g. facultative migrants [32,33]), therefore provides a novel framework for understanding the relationships between telomeres and behaviour.

Pine siskins (*Spinus pinus*) are seasonally breeding, nomadic songbirds that exhibit low site fidelity and a high degree of temporal and spatial flexibility in migratory behaviour [34,35]. Although they can migrate throughout the year, they frequently migrate in the spring to locate suitable habitat and opportunistically breed [34–36]. Captive individuals exhibit considerable variation in the timing and intensity of spring migratory restlessness [37,38], making them an excellent system to study the intrinsic causes and consequences of facultative migratory behaviour. To test the predictions of the causation and selective adoption hypotheses, we measured pre-migratory telomere lengths, telomere dynamics over the course of the migratory period, and the timing and intensity of spring migratory behaviour. We focus on male and female pine siskins experiencing their first spring migratory period to minimize effects of chronological age on behaviour [39,40].

## 2. Methods

### (a) Animals

Between June and November 2019, 35 wild, hatch year pine siskins were captured using mist nets or baited traps at multiple sites in Washington and Idaho and then housed at Washington State University (electronic supplementary material, §1). In captivity, birds were maintained on a naturally changing photoperiod (47° N latitude) and provided food, water and grit ad libitum. For this study, birds were housed indoors in individual cages. A ∼75 µl blood sample was collected from each bird using heparinized capillary tubes following brachial venipuncture on 13 February and 16 June 2020. Samples were immediately added to 750 µl of 100% ethanol and stored at −20°C until DNA extraction. Blood telomeres are useful biomarkers because they can be longitudinally sampled and relate to telomere lengths and dynamics of other tissues [41–43]. An aliquot of blood separated immediately after collection was sent to the Washington Animal Disease Diagnostic Laboratory for genetic sexing.

### (b) Behavioural data collection

Passive infrared sensors connected to a VitalView Data Acquisition System (Starr Life Sciences Corp., Oakmonk, PA, USA) measured nocturnal activity, an index of migratory restlessness and a predictor of migratory behaviour in free-living birds [37,44–46]. Activity was measured between 10 March 2020 and 9 May 2020, when birds express nocturnal migratory restlessness [37,38]. We did not focus on patterns of diurnal activity as pine siskins do not exhibit spring diurnal migratory restlessness [37,47]. Birds were housed in one of two rooms for the duration of behavioural data collection. We monitored 35 birds (14 females) for 69 nights for a total of 2415 observation-nights. We focus on two different dimensions of migratory behaviour: intensity and timing. The intensity of nocturnal activity was calculated by summing the total number of movements recorded between 23.00 and 03.00 of each night, a time window when birds exhibit migratory restlessness [37]. Timing of migratory initiation was assigned based on existing criteria [38]. A bird was classified as migratory on the first day of the first of three consecutive nights during which it exhibited six 10-minute bins of greater than 10 movements each. This threshold filters out isolated bouts of nocturnal activity to better capture sustained nocturnal activity characteristic of the onset of migratory restlessness. These criteria identified 13 of the 35 birds (six females) to have entered a migratory state (electronic supplementary material, figure S1), though birds below the threshold may exhibit some nocturnal activity. This ratio of birds classified as migratory is consistent with previous work wherein 17 of 49 individuals were classified migratory [38,47]. Because individuals were maintained in captivity for different amounts of time (range: 76–232 days before the experiment), we tested for an effect of days in captivity on telomere lengths, telomere dynamics, and migratory classification. Our analyses found no effect of days in captivity (electronic supplementary material, §2).

### (c) Relative telomere length measurement

DNA from whole blood samples was extracted using a Gentra Puregene Blood Kit (Qiagen) and the modified extraction protocol described in [23]. This extraction method as well as our approach for storing samples result in high molecular weight DNA suitable for telomere measurement by qPCR [23,48]. Relative telomere lengths (rTL) were quantified using real-time quantitative PCR (qPCR) following the methodologies of [48,49]. Glyceraldehyde-3-phoshate dehydrogenase (GAPDH) was used as the single-copy gene to quantify rTL. The quantification cycle (Cq) and individual well qPCR efficiencies for samples were calculated using *LINREGPCR* (version 11; [50]). rTL were calculated following equation one in [51] and *z*-transformed [52]. rTL technical repeatability was 0.7 (95% CIs = 0.36, 0.90). For more details, see electronic supplementary material, §3.

### (d) Statistical analyses

All statistical analyses were performed in R v. 3.6.2 [53]. We tested for sex-specific differences in rTL and telomere dynamics using linear mixed models. rTL was the response variable and the predictor variables included sex (mean centred), sampling time point, an interaction between sex and sampling time point, and a random intercept denoting individual ID. To determine if telomere dynamics depend upon migratory behaviour, we built two models. The first used the same model structure as above but sex was replaced with total amount of nocturnal activity observed over the course of the monitoring period. Nocturnal activity was log-transformed for model fit and then mean centred. In the second model, data were filtered to only birds classified as migratory and sex was replaced with mean centred migratory initiation date. Individual ID was nested within qPCR plate ID in the absence of model convergence issues. Repeatability of rTL was estimated using rptR [54]. We included a fixed, categorical effect of sampling date (February or June) and a random intercept term denoting individual ID nested within the qPCR plate. Bootstrapped 95% confidence intervals identified if repeatabilities were significantly different from zero. Repeatabilities were calculated with and without a male that exhibited a change in rTL that was 3 s.d. from the mean.

We built generalized linear mixed models with a negative binomial distribution using *glmmTMB* [55] to determine if pre-migratory telomere lengths or sex predict migratory behaviour. A quadratic polynomial term of experiment day was included to capture seasonal changes in nocturnal activity. All models also included a categorical, fixed effect denoting the room birds were housed in. A random intercept term denoting individual ID and a second degree polynomial random slope term were also included. Though this model is structurally complex, our dataset of 2415 observation nights allowed us to effectively estimate parameters. We checked model fit and assumptions using a simulation-based approach [56]. We quantified support for models including different interactive and additive effects of sex and rTL using AICc-based model selection [57]. To reduce model selection uncertainty, uninformative parameters were identified by examining whether 85% confidence intervals of predictor variables included 0 [58]. We used linear models and AICc-based model selection to assess the relationship between migratory initiation dates, rTL, and sex. We performed model diagnostics and assessed model fit for both models using the *check_model* function in performance package [59].

## 3. Results

rTLs were significantly repeatable, and across all individuals the repeatability estimate was 0.35 (95% CIs: 0.02, 0.61). Without the outlier male, the repeatability estimate was 0.51 (95% CI: 0.2, 0.71). rTL did not differ between the sexes (*β*_sex_ ± s.e. = 0.06 ± 0.35, *p* = 0.87) or sampling time points (*β*_June_ = 0.12 ± 0.17 *p* = 0.47). Telomere dynamics did not depend on sex (*β*_sex*time point_ = 0.26 ± 0.34, *p* = 0.45, electronic supplementary material, figure S2), total nocturnal activity (*β*_migratory activity*time point_ = 0.17 ± 0.12, *p* = 0.16, electronic supplementary material, figure S3*a*), or migratory initiation date (*β*_migratory date*time point_ = 0.001 ± 0.01, *p* = 0.93, electronic supplementary material, figure S3*b*).

**Figure 1.**
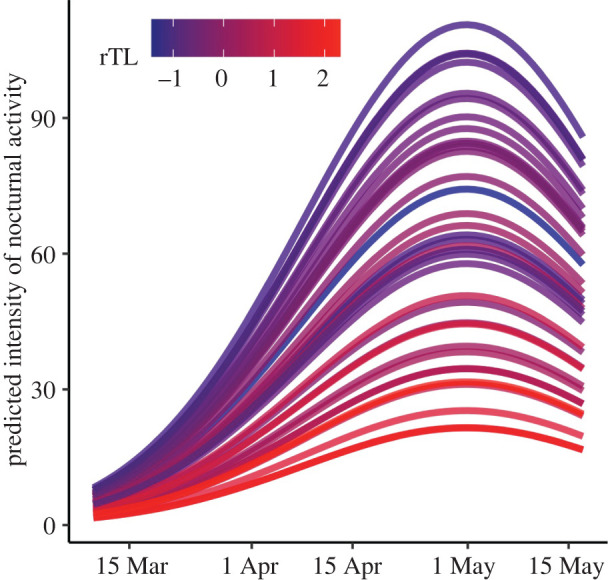
Predicted intensity of nocturnal activity (movements/4 h) during the spring migratory period calculated after setting random effects to zero using the model summarized in table 1*a*. Each line represents an individual and colour denotes differences in rTL (*n* = 35 birds).

**Figure 2.**
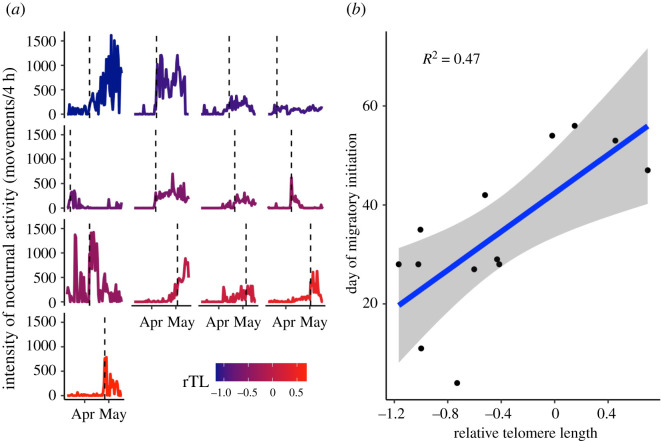
(*a*) Individual variation in intensity of nocturnal activity for migratory individuals during the spring migratory period. Vertical lines denote migratory initiation date and line colours denote differences in rTL. (*b*) rTL in relation to migratory initiation date (*n* = 13). Individuals are represented by filled circles. Blue lines and shaded areas depict the line of best fit and 95% confidence interval, respectively. Text inset is adjusted *R*^2^ of the top model.

Model selection identified a relationship between rTL and the intensity of nocturnal activity (figure 1; electronic supplementary material, table S1*a*). A model that included an additive effect of sex was similarly supported, but the 85% CIs identified sex as an uninformative parameter. The more parsimonious model revealed individuals with shorter rTL exhibited more intense nocturnal activity (figure 1 and table 1*a*). The top supported model explaining variation in migratory initiation date only included rTL and revealed shorter rTL to be associated with earlier migratory initiation dates (figure 2 and table 1*b*; electronic supplementary material, table S1*b*).

**Table 1.** Models used to examine the relationship between rTL and (*a*) the intensity of nocturnal migratory activity and (*b*) migratory initiation date.

Variable	estimate [95% CIs]	s.e.	*z*-value	*p*-value
(a) **Intensity of nocturnal activity**				
intercept	3.11 [2.75,3.48]	0.18	17.26	<0.0001
experiment day	33.65 [22.19, 45.12]	5.85	5.75	<0.0001
experiment day^2^	−16.86 [−24.77, −8.95]	4.04	−4.18	<0.0001
rTL	−0.36 [−0.60, −0.12]	0.12	−2.98	0.003
roomD	0.49 [0.03, 0.95]	0.24	2.09	0.04
(b) **Migratory initiation date**				
intercept	42.39 [33.48, 51.29]	4.04	10.48	<0.0001
rTL	19.45 [7.00, 31.9]	5.66	3.44	0.006

## 4. Discussion

Here, we show that the timing and intensity of migratory behaviour depends upon pre-migratory telomere lengths in a nomadic songbird. Individuals with shorter telomeres transitioned into a migratory state earlier and exhibited more intense nocturnal activity, independent of sex and, to some extent, chronological age. Our results support the selective adoption hypothesis since shorter, pre-migratory telomere lengths were associated with earlier, more intense migratory behaviour, and there was no evidence that telomere dynamics depended on an individual's behaviour. It remains to be determined whether selective adoption is driven by a direct effect of telomeres on behaviour or indirectly, via correlations between telomeres, behaviour, and a third factor (e.g. genetic makeup or early life adversity, [6]). Nonetheless, the intrinsic drivers of individual variation in migratory restlessness, a correlate of migratory behaviour in free-living migratory birds [37,44,46], are not well understood and our findings identify a strong link between telomeres and migratory restlessness. This adds to previous work indicating roles for fuelling dynamics [45,60], circadian rhythms [38] and variation in genes linked to circadian rhythms [61,62].

Telomere dynamics did not differ between the sexes or depend upon migratory behaviour. An important caveat here is that the combination of measurement error associated with qPCR-based measurements [63–65] and/or small sample size prevents us from completely ruling out an effect of migratory behaviour on telomere dynamics. This leaves open the possibility that both causation and selective adoption could operate simultaneously in this species [6]. Telomere dynamics have previously been used to understand somatic consequences of variable migratory behaviour [30,31], and our study reveals the potential for using telomeres to understand behavioural variation. Viewed together, there is strong potential for integrating studies of telomeres and migration to understand the causes and consequences of intra- and interspecific differences in migratory behaviour.

The timing of annual cycle events, including migration, can influence survival and reproduction [25–29,66,67]. Previous work in plants and animals has shown that reproductive timing depends upon telomere lengths [18,68,69]. By demonstrating that timing and intensity of the expression of migratory behaviour depends upon pre-migratory telomere lengths, we provide a new perspective on the consequences of telomere lengths and biological state [8] for the timing of annual cycle events and migratory decision-making. More broadly, this study adds to evidence highlighting the potential for telomeres to integrate information about an individual's physiology and environment and adaptively mediate behaviour [24,70].

## Data Availability

All data and code used to generate results are available from the Dryad Digital Repository: https://doi.org/10.5061/dryad.pk0p2ngqj [71].

## References

[1] McNamara JM, Houston AI. 1996 State-dependent life histories. Nature **380**, 215-221. (10.1038/380215a0) 8637568

[2] Ricklefs RE, Wikelski M. 2002 The physiology/life-history nexus. Trends Ecol. Evol. **17**, 462-468. (10.1016/S0169-5347(02)02578-8)

[3] Wingfield JC. 2013 Ecological processes and the ecology of stress: the impacts of abiotic environmental factors. Funct. Ecol. **27**, 37-44. (10.1111/1365-2435.12039)

[4] Stearns SC. 1992 The evolution of life histories. Oxford, UK: Oxford University Press.

[5] Houston AI, McNamara JM. 1999 Models of adaptive behaviour: an approach based on state. Cambridge, UK: Cambridge University Press.

[6] Bateson M, Nettle D. 2018 Why are there associations between telomere length and behaviour? Phil. Trans. R. Soc. B **373**, 20160438. (10.1098/rstb.2016.0438) 29335363 PMC5784059

[7] Tobler M, Gómez-Blanco D, Hegemann A, Lapa M, Neto JM, Tarka M, Xiong Y, Hasselquist D. In press. Telomeres in ecology and evolution: a review and classification of hypotheses. Mol. Ecol. (10.1111/mec.16308) 34865259

[8] Monaghan P. 2010 Telomeres and life histories: the long and the short of it. Ann. N. Y. Acad. Sci. **1206**, 130-142. (10.1111/j.1749-6632.2010.05705.x) 20860686

[9] Casagrande S, Hau M. 2019 Telomere attrition: metabolic regulation and signalling function? Biol. Lett. **15**, 20180885. (10.1098/rsbl.2018.0885) 30890069 PMC6451386

[10] Metcalfe NB, Olsson M. 2021 How telomere dynamics are influenced by the balance between mitochondrial efficiency, reactive oxygen species production and DNA damage. Mol. Ecol, 1-13. 34435398 10.1111/mec.16150

[11] von Zglinicki T. 2001 Stress, DNA damage and ageing — an integrative approach. Exp. Gerontol. **36**, 1049-1062. (10.1016/S0531-5565(01)00111-5) 11404050

[12] Angelier F, Costantini D, Blévin P, Chastel O. 2018 Do glucocorticoids mediate the link between environmental conditions and telomere dynamics in wild vertebrates? A review. Gen. Comp. Endocrinol. **256**, 99-111. (10.1016/j.ygcen.2017.07.007) 28705731

[13] Ilmonen P, Kotrschal A, Penn DJ. 2008 Telomere attrition due to infection. PLoS ONE **3**, e2143. (10.1371/journal.pone.0002143) 18478110 PMC2366059

[14] Tricola GM et al. 2018 The rate of telomere loss is related to maximum lifespan in birds. Phil. Trans. R. Soc. B **373**, 20160445. (10.1098/rstb.2016.0445) 29335369 PMC5784065

[15] Wilbourn RV, Moatt JP, Froy H, Walling CA, Nussey DH, Boonekamp JJ. 2018 The relationship between telomere length and mortality risk in non-model vertebrate systems: a meta-analysis. Phil. Trans. R. Soc. B **373**, 20160447. (10.1098/rstb.2016.0447) 29335371 PMC5784067

[16] Wang Q, Zhan Y, Pedersen NL, Fang F, Hägg S. 2018 Telomere length and all-cause mortality: a meta-analysis. Ageing Res. Rev. **48**, 11-20. (10.1016/j.arr.2018.09.002) 30254001

[17] Boonekamp JJ, Simons MJP, Hemerik L, Verhulst S. 2013 Telomere length behaves as biomarker of somatic redundancy rather than biological age. Aging Cell **12**, 330-332. (10.1111/acel.12050) 23346961

[18] Bauch C, Becker PH, Verhulst S. 2013 Telomere length reflects phenotypic quality and costs of reproduction in a long-lived seabird. Proc. R. Soc. B **280**, 20122540. (10.1098/rspb.2012.2540) PMC3574312 23222450

[19] Angelier F, Weimerskirch H, Barbraud C, Chastel O. 2019 Is telomere length a molecular marker of individual quality? Insights from a long-lived bird. Funct. Ecol. **33**, 1076-1087. (10.1111/1365-2435.13307)

[20] Bateson M, Brilot BO, Gillespie R, Monaghan P, Nettle D. 2015 Developmental telomere attrition predicts impulsive decision-making in adult starlings. Proc. R. Soc. B **282**, 20142140. (10.1098/rspb.2014.2140) PMC4286045 25473012

[21] Young RC, Kitaysky AS, Barger CP, Dorresteijn I, Ito M, Watanuki Y. 2015 Telomere length is a strong predictor of foraging behavior in a long-lived seabird. Ecosphere **6**, art39. (10.1890/ES14-00345.1)

[22] Adriaenssens B, Pauliny A, Blomqvist D, Johnsson JI. 2016 Telomere length covaries with personality in wild brown trout. Physiol. Behav. **165**, 217-222. (10.1016/j.physbeh.2016.07.005) 27470185

[23] Vernasco BJ, Dakin R, Majer AD, Haussmann MF, Brandt Ryder T, Moore IT. 2021 Longitudinal dynamics and behavioural correlates of telomeres in male wire-tailed manakins. Funct. Ecol. **35**, 450-462. (10.1111/1365-2435.13715)

[24] Young AJ. 2018 The role of telomeres in the mechanisms and evolution of life-history trade-offs and ageing. Phil. Trans. R. Soc. B **373**, 20160452. (10.1098/rstb.2016.0452) 29335379 PMC5784072

[25] Lerche-Jørgensen M, Korner-Nievergelt F, Tøttrup AP, Willemoes M, Thorup K. 2018 Early returning long-distance migrant males do pay a survival cost. Ecol. Evol. **8**, 11 434-11 449. (10.1002/ece3.4569) PMC6303765 30598747

[26] Price T, Kirkpatrick M, Arnold S. 1988 Directional selection and the evolution of breeding date in birds. Science **240**, 798-799. (10.1126/science.3363360) 3363360

[27] Smith RJ, Moore FR. 2005 Arrival timing and seasonal reproductive performance in a long-distance migratory landbird. Behav. Ecol. Sociobiol. **57**, 231-239. (10.1007/s00265-004-0855-9)

[28] Drent R, Both C, Green M, Madsen J, Piersma T. 2003 Pay-offs and penalties of competing migratory schedules. Oikos **103**, 274-292. (10.1034/j.1600-0706.2003.12274.x)

[29] Kokko H. 1999 Competition for early arrival in migratory birds. J. Anim. Ecol. **68**, 940-950. (10.1046/j.1365-2656.1999.00343.x)

[30] Schultner J, Moe B, Chastel O, Bech C, Kitaysky AS. 2014 Migration and stress during reproduction govern telomere dynamics in a seabird. Biol. Lett. **10**, 20130889. (10.1098/rsbl.2013.0889) 24429681 PMC3917333

[31] Bauer CM, Heidinger BJ, Ketterson ED, Greives TJ. 2016 A migratory lifestyle is associated with shorter telomeres in a songbird (*Junco hyemalis*). Auk **133**, 649-653. (10.1642/AUK-16-56.1)

[32] Hegemann A, Marra PP, Tieleman BI. 2015 Causes and consequences of partial migration in a passerine bird. Am. Nat. **186**, 531-546. (10.1086/682667) 26655576

[33] Newton I. 2012 Obligate and facultative migration in birds: ecological aspects. J. Ornithol. **153**, 171-180. (10.1007/s10336-011-0765-3)

[34] Dawson WR. 2020 Pine siskin (*Spinus pinus*), version 1.0. In Birds of the world (ed. AF Poole). Ithaca, NY: Cornell Lab of Ornithology. (10.2173/bow.pinsis.01)

[35] Cornelius JM, Hahn TP, Robart AR, Vernasco BJ, Zahor DL, Glynn KJ, Navis CJ, Watts HE. 2021 Seasonal patterns of fat deposits in relation to migratory strategy in facultative migrants. Front. Ecol. Evol. **9**, 1-14. (10.3389/fevo.2021.691808)

[36] Yunick RP. 1981 Some observations on the breeding status of the pine siskin. Kingbird fall **31**, 219-225.

[37] Watts HE, Robart AR, Chopra JK, Asinas CE, Hahn TP, Ramenofsky M. 2017 Seasonal expression of migratory behavior in a facultative migrant, the pine siskin. Behav. Ecol. Sociobiol. **71**, 9. (10.1007/s00265-016-2248-2)

[38] Rittenhouse JL, Robart AR, Watts HE. 2019 Variation in chronotype is associated with migratory timing in a songbird. Biol. Lett. **15**, 20190453. (10.1098/rsbl.2019.0453) 31455169 PMC6731483

[39] Ketterson ED, Nolan VJ, Nolan Jr. V. 1983 The evolution of differential bird migration. In Current ornithology, pp. 357-402. Berlin, Germany: Springer.

[40] Åkesson S, Bakam H, Martinez Hernandez E, Ilieva M, Bianco G. 2021 Migratory orientation in inexperienced and experienced avian migrants. Ethol. Ecol. Evol. **33**, 206-229. (10.1080/03949370.2021.1905076)

[41] Reichert S, Criscuolo F, Verinaud E, Zahn S, Massemin S. 2013 Telomere length correlations among somatic tissues in adult zebra finches. PLoS ONE **8**, e0081496. (10.1371/journal.pone.0081496) PMC3857187 24349076

[42] Asghar M, Palinauskas V, Zaghdoudi-Allan N, Valkiūnas G, Mukhin A, Platonova E, Färnert A, Bensch S, Hasselquist D. 2016 Parallel telomere shortening in multiple body tissues owing to malaria infection. Proc. R. Soc. B **283**, 20161184. (10.1098/rspb.2016.1184) PMC5013771 27488651

[43] Kesäniemi J et al. 2019 Exposure to environmental radionuclides associates with tissue-specific impacts on telomerase expression and telomere length. Sci. Rep. **9**, 850. (10.1038/s41598-018-37164-8) 30696885 PMC6351625

[44] Berthold P. 1973 Relationships between migratory restlessness and migration distance in six *Sylvia* species. Ibis (Lond. 1859). **115**, 594-599. (10.1111/j.1474-919X.1973.tb01998.x)

[45] Fusani L, Cardinale M, Carere C, Goymann W. 2009 Stopover decision during migration: physiological conditions predict nocturnal restlessness in wild passerines. Biol. Lett. **5**, 302-305. (10.1098/rsbl.2008.0755) 19324648 PMC2679912

[46] Schmaljohann H, Kämpfer S, Fritzsch A, Kima R, Eikenaar C. 2015 Start of nocturnal migratory restlessness in captive birds predicts nocturnal departure time in free-flying birds. Behav. Ecol. Sociobiol. **69**, 909-914. (10.1007/s00265-015-1902-4)

[47] Robart AR, McGuire MMK, Watts HE. 2018 Increasing photoperiod stimulates the initiation of spring migratory behaviour and physiology in a facultative migrant, the pine siskin. R. Soc. Open Sci. **5**, 180876. (10.1098/rsos.180876) 30225078 PMC6124035

[48] Eastwood JR, Mulder E, Verhulst S, Peters A. 2018 Increasing the accuracy and precision of relative telomere length estimates by RT qPCR. Mol. Ecol. Resour. **18**, 68-78. (10.1111/1755-0998.12711) 28805012

[49] Criscuolo F, Bize P, Nasir L, Metcalfe NB, Foote CG, Griffiths K, Gault EA, Monaghan P. 2009 Real-time quantitative PCR assay for measurement of avian telomeres. J. Avian Biol. **40**, 342-347. (10.1111/j.1600-048X.2008.04623.x)

[50] Ruijter JM, Ramakers C, Hoogaars WMH, Karlen Y, Bakker O, van den Hoff MJB, Moorman AFM. 2009 Amplification efficiency: linking baseline and bias in the analysis of quantitative PCR data. Nucleic Acids Res. **37**, e45-e45. (10.1093/nar/gkp045) 19237396 PMC2665230

[51] Pfaffl MW. 2001 A new mathematical model for relative quantification in real-time RT-PCR. Nucleic Acids Res. **29**, e45-e45. (10.1093/nar/29.9.e45) 11328886 PMC55695

[52] Verhulst S. 2020 Improving comparability between qPCR-based telomere studies. Mol. Ecol. Resour. **20**, 11-13. (10.1111/1755-0998.13114) 31680455 PMC6973063

[53] R Core Team. 2021 R: a language and environment for statistical computing. Vienna, Austria: R Foundation for Statistical Computing. See http://www.R-project.org.

[54] Stoffel MA, Nakagawa S, Schielzeth H. 2017 rptR: repeatability estimation and variance decomposition by generalized linear mixed-effects models. Methods Ecol. Evol. **8**, 1639-1644. (10.1111/2041-210X.12797)

[55] Brooks ME, Kristensen K, van Benthem KJ, Magnusson A, Berg CW, Nielsen A, Skaug HJ, Maechler M, Bolker BM. 2017 glmmTMB balances speed and flexibility among packages for zero-inflated generalized linear mixed modeling. R J. **9**, 378-400. (10.32614/RJ-2017-066)

[56] Hartig F. 2021 DHARMa: Residual Diagnostics for Hierarchical (Multi-Level / Mixed) Regression Models. *R Packag. version 0.4.4.*

[57] Burnham KP, Anderson DR, Huyvaert KP. 2011 AIC model selection and multimodel inference in behavioral ecology: some background, observations, and comparisons. Behav. Ecol. Sociobiol. **65**, 23-35. (10.1007/s00265-010-1029-6)

[58] Arnold TW. 2010 Uninformative parameters and model selection using Akaike's information criterion. J. Wildl. Manage. **74**, 1175-1178. (10.2193/2009-367)

[59] Lüdecke D, Ben-Shachar M, Patil I, Waggoner P, Makowski D. 2021 performance: an R package for assessment, comparison and testing of statistical models. J. Open Source Softw. **6**, 3139. (10.21105/joss.03139)

[60] Eikenaar C, Müller F, Kämpfer S, Schmaljohann H. 2016 Fuel accumulation advances nocturnal departure: a migratory restlessness study on northern wheatears at stopover. Anim. Behav. **117**, 9-14. (10.1016/j.anbehav.2016.04.017)

[61] Mueller JC, Pulido F, Kempenaers B. 2011 Identification of a gene associated with avian migratory behaviour. Proc. R. Soc. B **278**, 2848-2856. (10.1098/rspb.2010.2567) PMC3145181 21325325

[62] Peterson MP, Abolins-Abols M, Atwell JW, Rice RJ, Milá B, Ketterson ED. 2013 Variation in candidate genes *CLOCK* and *ADCYAP1* does not consistently predict differences in migratory behavior in the songbird genus *Junco*. F1000Research **2**, 115. (10.12688/f1000research.2-115.v1) 24627781 PMC3907158

[63] Nettle D, Gadalla SM, Lai T-P, Susser E, Bateson M, Aviv A. 2021 Measurement of telomere length for longitudinal analysis: implications of assay precision. Am. J. Epidemiol. **190**, 1406-1413. (10.1093/aje/kwab025) 33564874 PMC8245883

[64] Kärkkäinen T, Briga M, Laaksonen T, Stier A. In press. Within-individual repeatability in telomere length: a meta-analysis in nonmammalian vertebrates. Mol. Ecol. 1-21. (10.1111/mec.16155) 34455645

[65] Lindrose AR, McLester-Davis LWY, Tristano RI, Kataria L, Gadalla SM, Eisenberg DTA, Verhulst S, Drury S. 2021 Method comparison studies of telomere length measurement using qPCR approaches: a critical appraisal of the literature. PLoS ONE **16**, e0245582. (10.1371/journal.pone.0245582) 33471860 PMC7817045

[66] Thomas DW, Blondel J, Perret P, Lambrechts MM, Speakman JR. 2001 Energetic and fitness costs of mismatching resource supply and demand in seasonally breeding birds. Science **291**, 2598-2600. (10.1126/science.1057487) 11283370

[67] Post E, Forchhammer MC. 2008 Climate change reduces reproductive success of an Arctic herbivore through trophic mismatch. Phil. Trans. R. Soc. B **363**, 2367-2373. (10.1098/rstb.2007.2207) PMC2606787 18006410

[68] Bauer CM, Graham JL, Abolins-Abols M, Heidinger BJ, Ketterson ED, Greives TJ. 2018 Chronological and biological age predict seasonal reproductive timing: an investigation of clutch initiation and telomeres in birds of known age. Am. Nat. **191**, 777-782. (10.1086/697224) 29750556

[69] Choi JY et al. 2021 Natural variation in plant telomere length is associated with flowering time. Plant Cell **33**, 1118-1134. (10.1093/plcell/koab022) 33580702 PMC8599780

[70] Giraudeau M, Angelier F, Sepp T. 2019 Do telomeres influence pace-of-life-strategies in response to environmental conditions over a lifetime and between generations? Bioessays **41**, 1800162. (10.1002/bies.201800162) 30793350

[71] Vernasco BJ, Watts HE. 2022 Data from: Telomere length predicts timing and intensity of migratory behaviour in a nomadic songbird. Dryad Digital Repository. (10.5061/dryad.pk0p2ngqj) PMC9346355 35920029

